# Low-Shear Stress Promotes Atherosclerosis via Inducing Endothelial Cell Pyroptosis Mediated by IKKε/STAT1/NLRP3 Pathway

**DOI:** 10.1007/s10753-023-01960-w

**Published:** 2024-02-05

**Authors:** Yifei Lv, Zihao Jiang, Wenying Zhou, Hongfeng Yang, Guozhen Jin, Dongchen Wang, Chaohua Kong, Zhiyuan Qian, Yue Gu, Shaoliang Chen, Linlin Zhu

**Affiliations:** 1 Department of Cardiology, Nanjing First Hospital, Nanjing Medical University, Nanjing, Jiangsu 210000 China; 2 Department of Intensive Care Unit, Affiliated People’s Hospital of Jiangsu University, Zhenjiang, Jiangsu 212000 China

**Keywords:** low-shear stress, pyroptosis, IKKε, STAT1, NLRP3, atherosclerosis

## Abstract

Atherosclerosis is initiated by vascular endothelial dysfunction, and low-shear stress (LSS) of blood flow is a key factor leading to endothelial dysfunction. Growing evidence suggests that endothelial cell pyroptosis plays an important role in the development of atherosclerosis. Studies have shown that low-shear stress can induce endothelial cell pyroptosis, but the exact mechanism remains unclear. Our experiments demonstrated that low-shear stress induced endothelial cell pyroptosis and the phosphorylation of IκB kinase ε (IKKε). IKKε knockdown not only significantly attenuated atherosclerosis lesions of aortic arch areas in ApoE^−/−^ mice fed with high cholesterol diets, but also markedly reduced endothelial cell pyroptosis and NLRP3 expression triggered by low-shear stress. Further mechanism studies showed that IKKε promoted the expression of NLRP3 via activating signal transducer and activator of transcription 1 (STAT1) and the subsequent binding of STAT1 to NLRP3 promoter region. These results suggest that low-shear stress plays a pro-atherosclerotic role by promoting endothelial cell pyroptosis through the IKKε/STAT1/NLRP3 pathway, which provides new insights into the formation of atherosclerosis.

**Supplementary Information:**

The online version contains supplementary material available at 10.1007/s10753-023-01960-w.

## INTRODUCTION

Atherosclerosis preferentially occurs in the medial curvature and bifurcation of vessels, which is closely related to changes in local blood flow patterns, especially low-shear stress (LSS) [1]. At the cellular and mechanistic levels, in contrast to pulsatile shear stress (PSS) in straight blood vessels, low-shear stress promotes inflammatory phenotype switching and death of endothelial cells, leading to the early onset of atherosclerosis [2].

Differently from apoptosis and necrosis, pyroptosis is a novel type of inflammatory programmed cell death characterized by NLRP3 inflammasome-dependent activation of caspase-1 which is responsible for the maturation of the precursor Gasdermin D (GSDMD), interleukin-1β (IL-1β), and interleukin-18 (IL-18), ultimately inducing cell membrane rupture as well as the release of mature IL-1β and IL-18 [3]. Mounting studies have shown that endothelial cell pyroptosis plays a crucial role in cardiovascular diseases including atherosclerosis, myocardial infarction, and diabetic cardiomyopathy [4]. Emerging studies found that low-shear stress upregulated NLRP3 expression through the ROS/NFκB pathway and participated in endothelial cell inflammatory phenotype switching, suggesting that low-shear stress mediates endothelial cell pyroptosis [5–7]. However, the mechanism by which low-shear stress induces endothelial cell pyroptosis remains unclear.

IκB kinase ε (IKKε), a non-canonical member of the IKK family, acts as a key regulator of innate or adaptive immunity. IKKε not only mediates lipopolysaccharide-induced inflammation by activating transcription factor nuclear factor κB (NFκB) P65 or signal transducer and activator of transcription 1 (STAT1), but also regulates type I interferon signaling by promoting the phosphorylation of interferon regulatory factor 3 (IRF3) [8]. Meanwhile, our previous studies demonstrated that phosphorylated IKKε in response to low-shear stress mediated endothelial inflammation by activating STAT1 and Akt/IRF3 pathways [9, 10]. However, the role and mechanism of IKKε in endothelial cell pyroptosis in response to low-shear stress are poorly understood.

The aim of this study was to explore the role of IKKε in LSS-induced endothelial cell pyroptosis and atherosclerosis. Both *in vitro* and *in vivo* experiments showed that the phosphorylation level of IKKε was significantly increased under low-shear stress. At the cellular level, silencing IKKε significantly reduced LSS-induced endothelial cell NLRP3 expression and pyroptosis via inhibiting the activation of STAT1 and the subsequent binding of STAT1 to the NLRP3 promoter region. Similarly, mice experiments showed that endothelial-specific knockdown of IKKε reduced NLRP3 expression and alleviated aortal arch atherosclerotic lesions.

## MATERIALS AND METHODS

### Cell Culture and Treatments

Human umbilical vein endothelial cells (HUVECs) were purchased from the Cell Bank of the Chinese Academy of Sciences (Shanghai, China). The cells were cultured in endothelial cell medium (ECM, ScienCell Research Laboratories, Carlsbad, CA, USA) supplemented with 10% fetal bovine serum (FBS), and 1% (v/v) penicillin/streptomycin at 37 °C with 5% CO_2_ air. When cells grow to 40–60% confluence, transfect siRNA and plasmid with Lipofectamine 3000 (Thermo Fisher Scientific, L3000001) according to the instructions. Cells were stimulated with shear stress after 48 h of culture. All siRNAs mentioned in the article were purchased from GenePharma (Shanghai, China). Target siRNA sequences are shown in Table S1.

### Shear Stress Experiments *In Vitro*

In order to simulate the *in vitro* shear stress experiment, the HUVECs were seeded on glass slides in advance, and after cell transfection or pharmacological treatment, the slides were moved into the flat flow chamber system (NatureThink, Shanghai, China), and DMEM containing 10% fetal bovine serum was injected into the chamber to cover endothelial cells. A unidirectional rate controller regulates the flow rate and exposes the endothelial cells on the slide to LSS (2 dyne/cm^2^) or PSS (15 dyne/cm^2^) for 8 h.

### Western Blot Analysis

The total protein of HUVECS was extracted using RIPA-containing protease inhibitors and phosphatase inhibitors. After the protein concentration was quantified, the same amount of protein (30 μg) was separated by 10% SDA-PAGE gel and transferred to the PVDF membrane. After blocking with 5% non-fat milk in TBST for 2 h, the membrane was incubated with the corresponding primary antibody overnight. Membranes were blocked with 5% bovine serum albumin in TBST for 2 h and then incubated overnight with corresponding primary antibodies at appropriate dilution ratios:IKKε (1:1000, Abcam, ab7891), p-IKKε Ser172 (1:1000, CST, #8766), STAT1 (1:1000, Abcam, ab234400), p-STAT1 Tyr701 (1:1000, CST, #7649), P65 (1:1000, CST, #8242), p-P65 Ser536 (1:1000, CST, #3033), p-IκBα Ser32 (1:1000, CST, #2859), NLRP3 (1:1000, Bioworld, BS90949), caspase-1 (1:1000, Proteintech, 22915-1-AP), GSDMD (1:1000, CST, #39754), IL-1β (1:1000, CST, #83186), IL-18 (1:1000, CST, #54943), β-actin (1:1000, CST, #3700), Histone H3 (1:1000, CST, #4499). Membranes were incubated with corresponding HRP-conjugated secondary antibodies for 2 h at room temperature after washing with TBST. Protein bands were visualized using an ECL chemiluminescent solution (Yeasen, China, Cat# 36208ES60) and the intensity of bands was quantified using NIH ImageJ software 1.43.

### RNA Isolation and Quantitative Real-Time PCR

Total RNA from collected endothelial cells were extracted using Trizol (Vazyme, China, Cat# R701). The relative expression of mRNA was measured by amplifying the first strand of cDNA by the ChamQ Universal SYBR qPCR Master Mix (Vazyme, China, Cat# Q711-02) after cDNA was obtained using RNA reverse transcribed by the HiScript III 1st Strand cDNA Synthesis Kit (Vazyme, China, Cat# R312-01/02). The relative gene expression levels were calculated using the 2^−△△CT^ method and 18 s was used as a normalized reference gene. All primer sequences are shown in Table S2.

### Modeling Atherosclerosis and Ethical Statement

All C57BL/6 mice and ApoE^−/−^ mice (C57BL/6 background) in this experiment were purchased from Gempharmatech, Nanjing, China. All mice were housed in a pathogen-free standard housing environment. Endothelial-specific IKKε knockdown adeno-associated virus (AAV) was purchased from BrainVTA (Wuhan, China). After construction of rAAV-tie2-IKKε(IKKε^KD^) by inserting IKKε shRNA into rAAV-tie2-mCherry-5′miR-30a-shRNA (IKKε)-3′miR-30a-WPREs, 8-week-old ApoE^−/−^ mice were randomly divided into 2 groups of 6 mice each. One group was injected with 100 µl of rAAV-tie2-IKKε at a viral titer of 2 × 10^12^ genomes per ml via the tail vein, and the other group was injected with an equal dose of rAAV-tie2-vectors (IKKε^vector^). Two groups were fed a high cholesterol diet (HCD) for 12 weeks after 4 weeks on a normal diet. The aortic arch and descending aorta of mice were extracted after euthanasia for further experiments. All animal protocols were approved by the Committee on the Ethics of Animal Experiments of Nanjing Medical University. Human coronary artery tissues were collected from dilated cardiomyopathy patients undergoing heart transplantation. All experimental protocols conformed to the Declaration of Helsinki and were supported by the Ethics Committee of Nanjing Medical University. All patients obtained written informed consent.

### Enzyme-Linked Immunosorbent Assay (ELISA)

The human IL-1β ELISA kit was purchased from ABclonal (Wuhan, China, Cat# RK00001). After HUVECs were exposed to LSS or PSS stimulation for 8 h, undiluted cell supernatants were collected and the levels of IL-1β secreted by the cells were measured according to the manufacturer's instructions.

### Cell Death Assay

Pyroptotic death of HUVECs was assessed using the detection of lactate dehydrogenase (LDH) content in cell supernatant and Hoechst 33342/PI staining. The LDH assay kit was purchased from Jiancheng Biology Engineering Institute (Nanjing, China, Cat#A020-2-2). Briefly, the collected cell supernatant was mixed with the substrate and incubated at 37 ℃ for 15 min, after which 2,4‐dinitrophenylhydrazine was added and incubated for 15 min under the same conditions, and finally, NaOH was added to terminate the reaction. The absorbance was detected at 450 nm using an enzyme standardization instrument. Hoechst 33,342/PI double stain kit was purchased from Yeasen Institute of Biotechnology (Shanghai, China, Cat#40744ES60). According to the manufacturer’s instructions, the treated cells were sequentially subjected to Hoechst 33342/PI double staining and then observed under a fluorescent microscope (Olympus, FV300).

### TUNEL Staining

After shear stimulation or pharmacological treatment, cells were fixed with paraformaldehyde and then washed 3 times with PBS. After immersion in 0.1% Triton X-100 for 15 min, the cells were treated with a TUNEL staining kit (Yeasen, Shanghai, China, Cat#40308ES60) according to the manufacturer’s instructions. Cell images were obtained using fluorescence microscopy (Olympus, FV300).

### Immunofluorescence Staining

Mouse vascular tissues and endothelial cells were permeabilized with 0.1% Triton X-100 for 15 min after fixation with paraformaldehyde. Samples were blocked with 5% bovine serum albumin for 2 h and then incubated with anti-p-IKKε Ser172 (1:200, Bioss,8583R), anti-IKKε (1:200, Abcam, ab7891), anti-p-STAT1 Tyr701 (1:200, CST, #9167), STAT1 (1:1000, Abcam, ab234400), anti-NLRP3 (1:200, Bioworld, BS90949), and anti-vWF (1:200, Proteintech, 66682-1-Ig) primary antibodies overnight. All samples were incubated with the corresponding fluorescent-coupled secondary antibody for 2 h at room temperature, and then nuclei were stained with DAPI. Images were acquired using laser scanning confocal microscopy (Carl Zeiss, LSM 710).

### Histological Staining

Hematoxylin-eosin (HE) staining was used to assess the extent of atherosclerotic plaque lesions, and Oil red O staining was used to assess the intra-plaque lipid content. The mouse aorta was stripped and fixed overnight with paraformaldehyde before optimum cutting temperature compound (OCT) embedding. Tissue sectioning was followed by HE staining using standard protocols. The mouse aorta was incubated with Oil red O reagent for 30 min and then decolorized with isopropyl alcohol. Images were acquired under the microscope (Olympus, BX43).

### Chromatin Immunoprecipitation (ChIP) Assay

The ChIP assay kit (Abcam, UK, ab500) was used to detect the presence of binding of transcription factors to potential promoter regions of target genes. Briefly, the anti-STAT1 (1:500, Abcam, ab234400) primary antibody is conjugated to magnetic beads and incubated with cell lysate to precipitate the DNA-protein complex. The DNA was purified and amplified by polymerase chain reaction (PCR) using primers designed with binding sites predicted by the JASPAR database. PCR amplification products were detected using agarose electrophoresis. The primer sequences of all predicted binding sites are shown in Table S3.

### Statistical Analysis

Statistical analysis was performed using GraphPad Prism 8.0 software. All results are expressed as mean ± SEM. Unpaired *t* test was used for comparison between two groups, and one-way analysis of variance (ANOVA) was used for comparison between multiple groups. A *p*-value less than 0.05 was considered statistically significant.

## RESULTS

### Low-Shear Stress Triggers Endothelial Cell Pyroptosis and IKKε Activation *In Vitro *and *In Vivo*

**Fig. 1:**
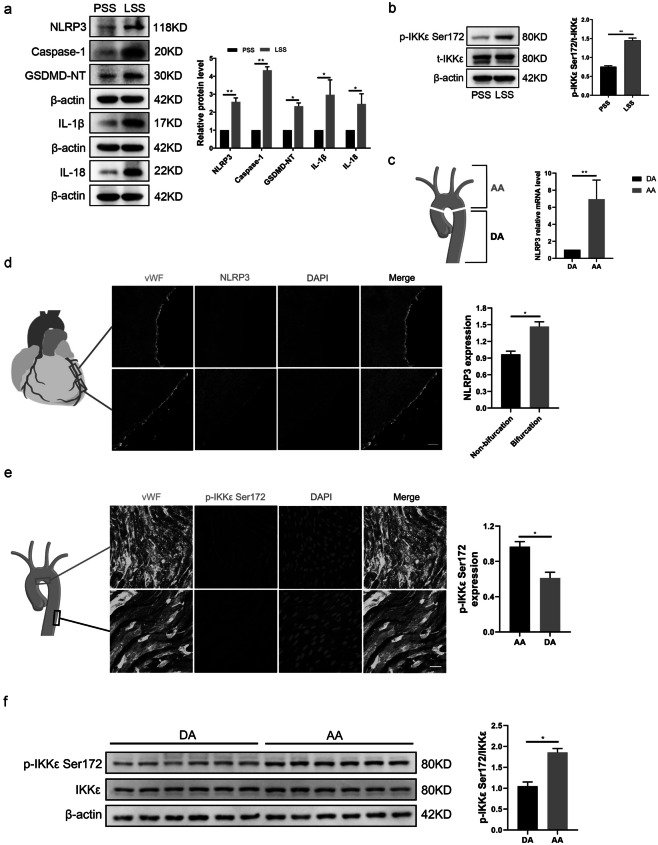
LSS triggers endothelial cell pyroptosis and IKKε activation *in vitro* and *in vivo*. **a**, **b** Validation of changes in pyroptosis-related protein (caspase-1, GSDMD-NT, NLRP3, IL-1β, IL-18) and IKKε phosphorylation levels in HUVECs after 8 h exposure to PSS/LSS by western blot (*n* = 3). **c** Detection of NLRP3 expression in the intima of the aortic arch (AA) and descending aorta (DA) from C57BL/6 mice (*n* = 6). **d** NLRP3 immunofluorescence (red) in the intima of bifurcation and non-bifurcation regions of human coronary arteries (*n* = 3). Scale bar = 200 μm. **e** Enface staining demonstrated the phosphorylation level of IKKε Ser172 (red) in the intima of mouse aortic arch medial bend and descending aorta (*n* = 3). Scale bar = 20 μm. **f** Detection of phosphorylation level of IKKε in the intima of the descending aorta (DA) and aortic arch (AA) from C57BL/6 mice (*n* = 6). The data are presented as mean ± SEM. ^*^p < .05, ^**^p < .01.

Previous studies have shown that in straight blood vessels such as the thoracic aorta, physiological pulsatile shear stress (PSS) is dominant, with a range of 15–70 dyne/cm^2^. On the contrary, low-shear stress (LSS) tends to occur at the inner bends and bifurcations of curved blood vessels. The approximate range is < 10–12 dyne/cm^2^ [7, 11, 12]. To investigate the effect of LSS on endothelial cell pyroptosis and IKKε phosphorylation, we exposed HUVECs to LSS/PSS for 8 h and then performed Western blot, which showed that LSS significantly upregulated pyroptosis-related protein NLRP3, cleavage of caspase-1, GSDMD-NT, and mature pro-inflammatory factor IL-1β and IL-18 (Fig. 1a). Meanwhile, LSS triggered the phosphorylation of IKKε Ser172, which was consistent with our previous findings [10] (Fig. 1b). Furthermore, we examined mRNA levels of pyroptosis-related molecules in the endothelium of the aortic arch (AA) and descending aorta (DA) in C57BL/6 mice and results showed that the expression of NLRP3 evidently increased in the aortic arch area (Fig. 1c). We further examined NLRP3 expression in the intima of human coronary arteries at different locations using immunofluorescence. The fluorescence intensity of NLRP3 was more pronounced at the coronary bifurcation where low-shear stress tends to occur, compared to the intima of the non-bifurcation coronary artery (Fig. 1d). Meanwhile, enface staining revealed that the intimal IKKε phosphorylation level in the medial bend of the mouse aortic arch was significantly higher than that in the descending aorta (Fig. 1e). Consistently, we isolated the intima of the descending aorta and aortic arch from C57BL/6 mice. Western blot showed that the phosphorylation level of IKKε in the aortic arch intima was significantly higher than that in the descending aorta (Fig. 1f). These results indicate that LSS triggers endothelial cell pyroptosis and activates IKKε.

### Adeno-associated Virus-Mediated Endothelial-Specific IKKε Knockdown Alleviates Atherosclerotic Lesions

**Fig. 2:**
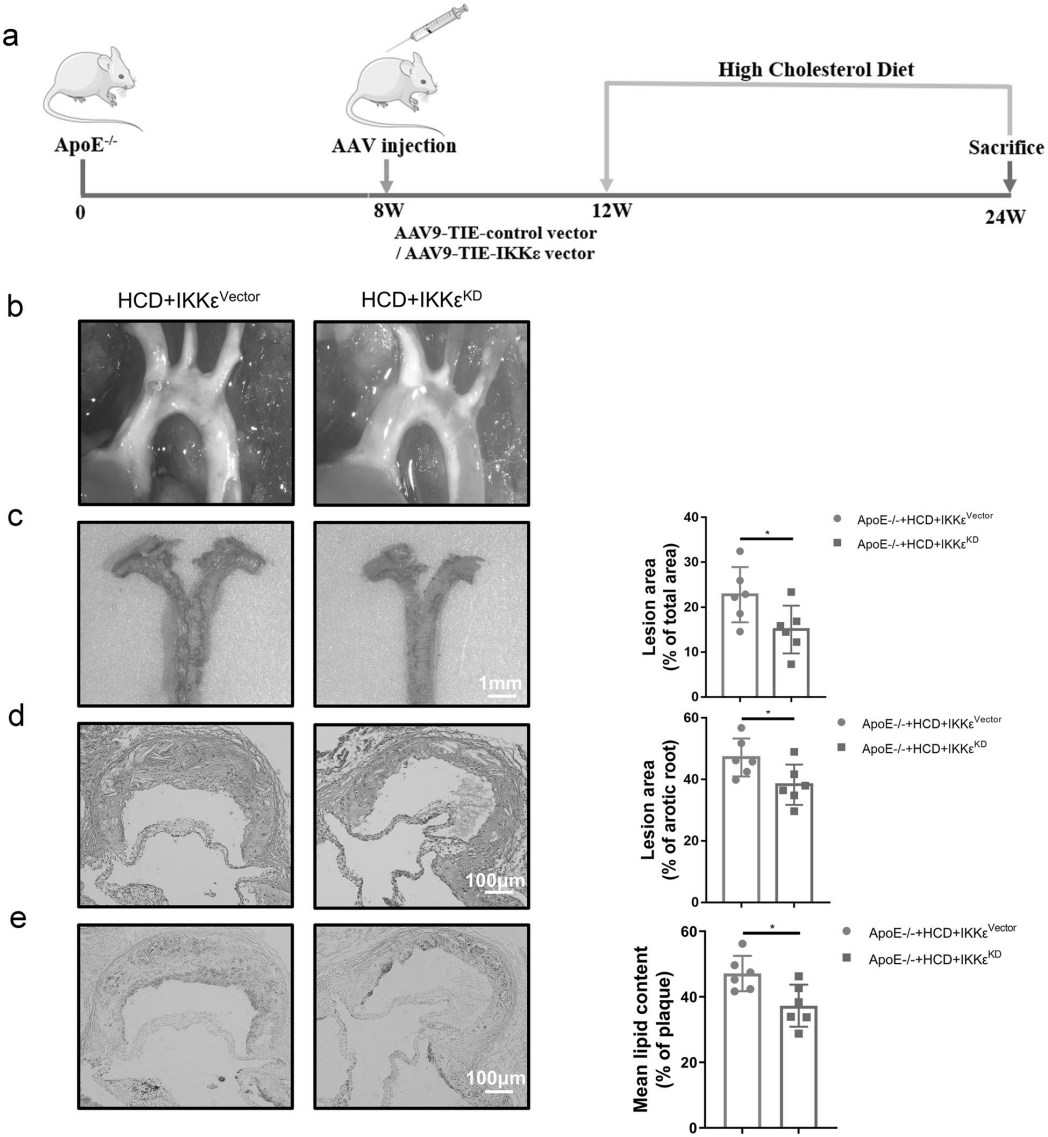
Adeno-associated virus-mediated endothelial-specific IKKε knockdown alleviates atherosclerotic lesions. ApoE^−/−^ mice were randomly divided into 2 groups of 6 mice each: HCD+IKKε^vector^ or HCD+IKKε^KD^. **a** Schematic diagram of the construction and treatment of mouse atherosclerosis model. **b** Image of the overall area of the aortic arch plaque using a dissecting microscope. **c** Oil red O staining of isolated aortic arch, the right histogram is a quantification of the plaque area in the left image (*n* = 6). Scale bar = 1 mm. **d**, **e** Representative images of HE staining and Oil red O staining of the aortic root, demonstrating the area and lipid content of atherosclerotic plaques. The bar graph on the right is a quantification of the corresponding staining (*n* = 6). Scale bar = 100 μm. The data are presented as mean ± SEM. ^*^p < .05, ^**^p < .01.

To explore the effect of IKKε activation on LSS-induced atherosclerotic lesions, especially in the medial bend and bifurcation of the aortic arch, we constructed an HCD-induced atherosclerosis model with or without endothelial-specific IKKε knockdown by administering rAAV-tie2-IKKε (IKKε^KD^) or rAAV-tie2-vectors (IKKε^vector^) adeno-associated virus in ApoE^−/−^ mice (Fig. 2a). First, aorta enface staining demonstrated that endothelial-specific knockdown of IKKε was effective (Fig. S1A). Next, the body weight of each mouse in each group was recorded weekly, and results showed no significant difference in mouse body weight between the two groups (Fig. S1B). In aortic arch segments, the extent and scope of atherosclerotic lesions were found to be significantly reduced in the IKKε^KD^ group compared with the IKKε^vector^ group under the dissecting microscopy (Fig. 2b). Consistent with this, Oil red O staining showed that endothelial-specific knockdown of IKKε alleviated atherosclerosis, especially at aortic arch bifurcations and medial bends (Fig. 2c). Aortic sinus sections were stained with HE and Oil red O, and results declared significant reductions in the area and lipid content of atherosclerotic plaques after specific knockdown of endothelial IKKε (Fig. 2d, e). In summary, all data suggest that endothelial-specific IKKε knockdown alleviates atherosclerotic lesions in the HCD-induced atherosclerosis model, especially in the LSS region.

### IKKε Is Involved in LSS-Triggered Endothelial Cell Pyrotosis

**Fig. 3:**
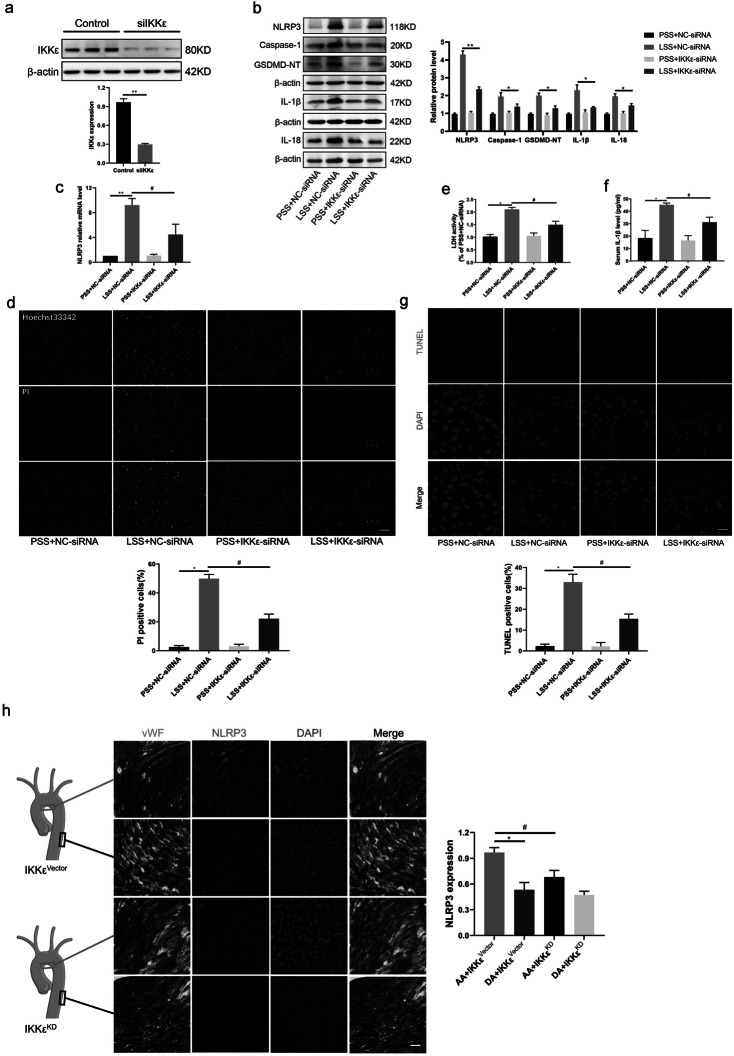
IKKε is involved in LSS-triggered endothelial cell pyrotosis. **a** The effectiveness of IKKε-siRNA knockdown on IKKε was detected by western blot. **b**, **c** Detection effects of IKKε silencing on pyroptosis-related molecules at protein level (caspase-1, GSDMD-NT, NLRP3, IL-1β, IL-18) and NLRP3 mRNA level using western blot and RT-qPCR (*n* = 3). **d** The percentage of PI (red) positive cells were decreased in LSS-treated HUVECs after transfection with IKKε-siRNA (*n* = 3). Scale bar = 200 μm. **e**, **f** Changes in IL-1β and LDH content in the supernatant of LSS-treated endothelial cells with or without transfection with IKKε-siRNA were detected using ELISA and LDH kits (*n* = 3). **g** TUNEL staining showed the proportion of TUNEL-positive (red) cells in the field of view with or without IKKε silencing in different fluid patterns (*n* = 3). Scale bar = 20 μm. **h** Enface staining revealed that intimal NLRP3 fluorescence intensity of the aortic arch medial bend (LSS region) significantly decreased in the IKKε^KD^ group, compared with the IKKε^vector^ group (*n* = 3). Scale bar = 20 μm. The data are presented as mean ± SEM. ^*^p < .05, ^**^p < .01, ^#^*p* < .05.

To clarify the role of IKKε in LSS-triggered endothelial cell pyroptosis, we first transfected IKKε siRNA to knockdown IKKε before detecting the level of endothelial cell pyroptosis in response to low-shear stress (Fig. 3a). Results revealed that knockdown of IKKε reversed LSS-triggered high expression of pyroptosis-associated protein NLRP3, cleavage of caspase-1 and GSDMD-NT, and IL-1β as well as IL-18 (Fig. 3b). Moreover, elevated mRNA levels of NLRP3 in response to LSS significantly downregulated after IKKε silencing (Fig. 3c). Knockdown of IKKε also decreased the cell membrane disruption indicated by Hoechst33342/PI staining and levels of LDH in cell supernatants (Fig. 3d, e). In addition, IL-1β content in the supernatant of LSS-treated HUVECs detected using ELISA was significantly reduced in the IKKε silencing group (Fig. 3f). TUNEL staining also showed a consistent trend (Fig. 3g). At the animal level, enface staining revealed that intimal NLRP3 fluorescence intensity of the aortic arch medial bend (LSS region) significantly decreased in the IKKε^KD^ group, compared with the IKKε^vector^ group (Fig. 3h). Both *in vitro* and *in vivo* experiments reveal that IKKε is closely involved in LSS-triggered endothelial cell pyroptosis.

### IKKε Activates Downstream Transcription Factor STAT1 in LSS-Treated HUVECs

**Fig. 4:**
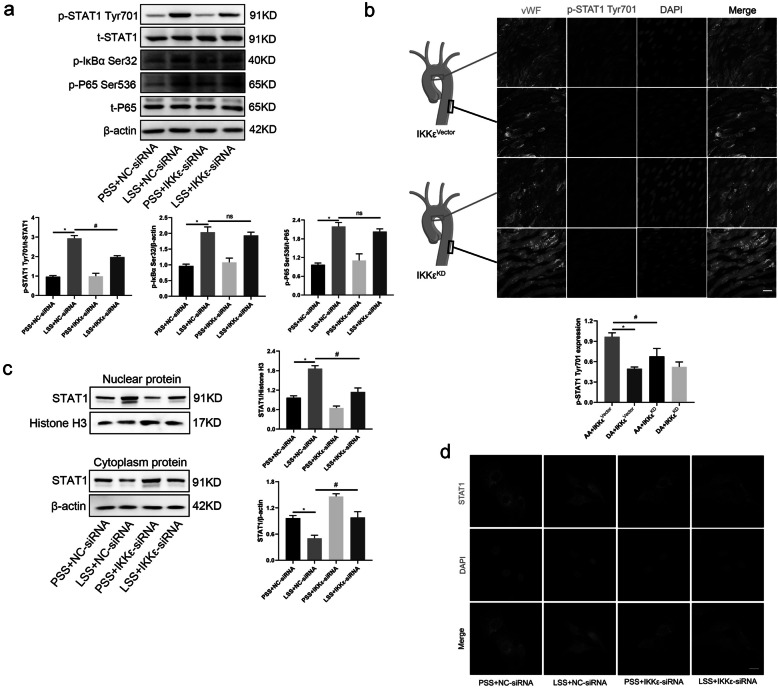
IKKε activates transcription factor STAT1. **a** Detection of the effects of IKKε silencing on the phosphorylation of STAT1, IκBα, and NFκB P65 by western blot (*n* = 3). **b** Enface staining of STAT1Tyr701 in the aortic arch and descending aorta (red) of IKKε^KD^ or IKKε^vector^ mice (*n* = 3). Scale bar = 20 μm. **c**, **d** Western blot assayed STAT1 levels in cytoplasm and nucleus of endothelial cells with or without IKKε silencing under LSS stimulation. Immunofluorescence was used to observe the distribution of STAT1 (red) in the cytoplasm and nucleus under the same conditions (*n* = 3). Scale bar = 10 μm. The data are presented as mean ± SEM. ^*^p < .05, ^#^*p* < .05.

To clarify the specific mechanism of IKKε-mediated endothelial cell pyroptosis in response to LSS, we further explored its potential downstream molecules including STAT1, IκBα, and NFκB P65. Phosphorylation levels of STAT1 and IκBα/NFκB were simultaneously elevated under LSS treatment, but in the case of IKKε silencing, only STAT1 phosphorylation was significantly inhibited, rather than IκBα and NFκB P65 (Fig. 4a). *In vivo*, enface staining revealed that intimal STAT1 phosphorylation of the aortic arch medial bend was significantly reduced in the endothelial-specific IKKε^KD^ group, compared with the IKKε^vector^ group (Fig. 4b). Furthermore, silencing of IKKε reversed LSS-mediated nuclear translocation of STAT1 assessed by Western blot and immunofluorescence assay (Fig. 4c, d). Thus, these results suggest that STAT1 acts as a downstream molecule of IKKε in LSS-treated HUVECs.

### STAT1 Mediates LSS-Triggered Endothelial Pyrotosis via Binding to the Promoter Region of NLRP3

**Fig. 5:**
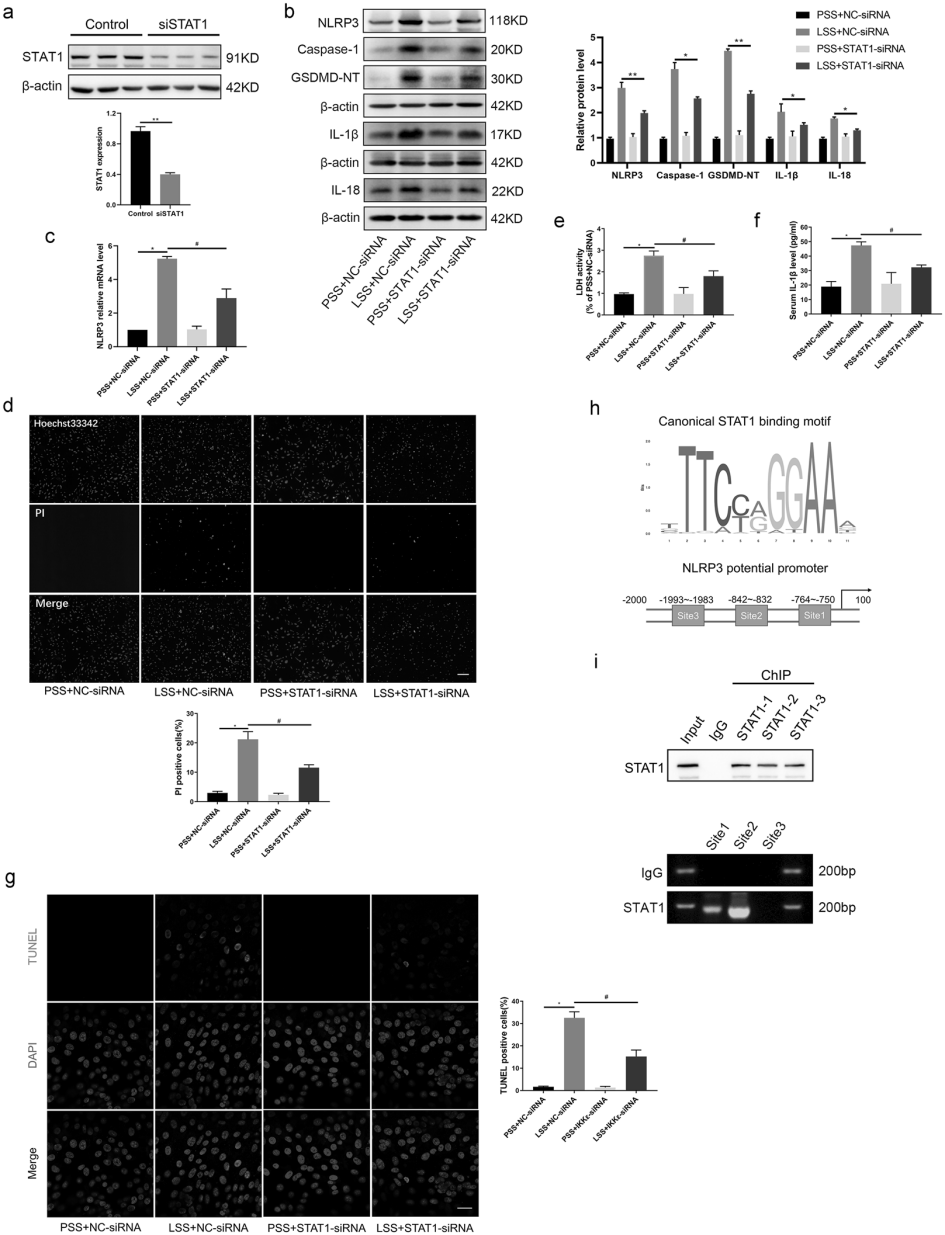
STAT1 mediates LSS-triggered endothelial pyrotosis via binding to the promoter region of NLRP3. **a** The effectiveness of IKKε-siRNA knockdown on IKKε was detected by western blot. **b**, **c** Detection effects of STAT1 silencing on pyroptosis-related molecules at protein level (caspase-1, GSDMD-NT, NLRP3, IL-1β, IL-18) and NLRP3 mRNA level using western blot and RT-qPCR (*n* = 3). **d** The percentage of PI (red) positive cells was decreased in LSS-treated HUVECs after transfection with STAT1-siRNA (*n* = 3). Scale bar = 200 μm. **e**, **f** In the supernatant of LSS-treated HUVECs with or without STAT1-siRNA transfection, levels of IL-1β and LDH were measured by ELISA and LDH kit (*n* = 3). **g** TUNEL staining showed the proportion of TUNEL-positive (red) cells with or without STAT1 silencing in different fluid patterns (*n* = 3). Scale bar = 20 μm. **h**, **i** The JASPAR database predicted the potential binding sites of STAT1 on NLRP3 promoter region and confirmed the binding site using ChIP experiments (*n* = 3). The data are presented as mean ± SEM. ^*^p < .05, ^**^p < .01, ^#^*p* < .05.

We further investigated the effect of IKKε-mediated STAT1 activation on endothelial cell pyroptosis in response to LSS. We first transfected STAT1 siRNA to knockdown STAT1 (Fig. 5a). Western blot showed that silencing of STAT1 reversed LSS-triggered expression of NLRP3, cleavage of caspase-1 and GSDMD-NT, and IL-1β and IL-18 in HUVECs (Fig. 5b). Similarly, the silencing of STAT1 significantly decreased mRNA expression levels of NLRP3 in response to LSS (Fig. 5c). Moreover, STAT1 silencing evidently reduced the number of PI-positive cells and the level of LDH in the supernatant of LSS-treated HUVECs by using Hoechst33342/PI assay and LDH kit (Fig. 5d, e. Silencing of STAT1 alleviated high levels of IL-1β in cell supernatants due to LSS (Fig. 5f). TUNEL staining demonstrated that the number of positive cells apparently decreased in STAT1-silencing groups (Fig. 5g). To explore whether STAT1 promoted NLRP3 expression via binding to its promoter region, we utilized the JASPAR database to predict and achieved 3 potential binding sites: site 1 (−764 ~−750), site 2 (−842~−832), site 3 (−1993~−1983) (Fig. 5h). We further validated these sites with ChIP experiments which showed that STAT1 binds to site 1 and site 2 of the NLRP3 promoter region (Fig. 5i). Thus, STAT1 promotes LSS-induced NLRP3 expression and endothelial pyrotosis via binding to the promoter region of NLRP3.

## DISCUSSION

**Fig. 6:**
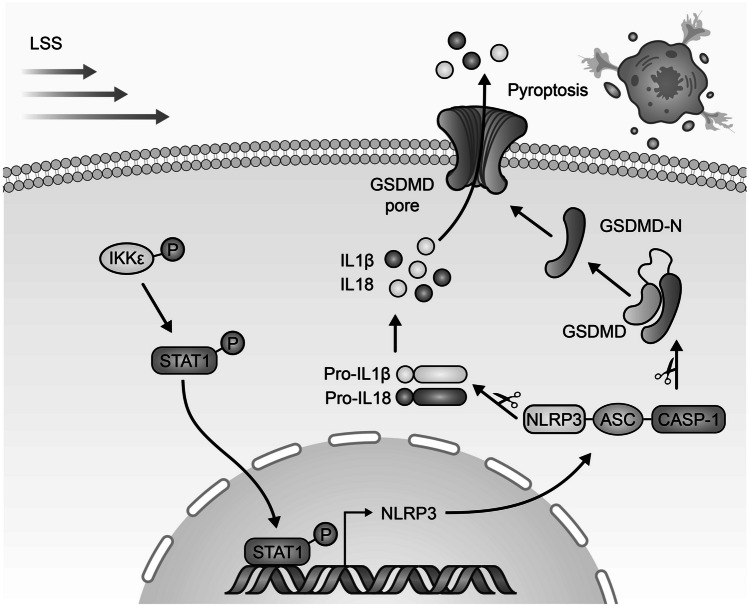
LSS affects endothelial cell pyroptosis through IKKε/STAT1/NLRP3 signaling pathway. Activation of IKKε induced by LSS triggers the nuclear translocation of STAT1 and the subsequent binding of STAT1 to NLRP3 promoter region, promoting the expression of NLRP3 and formation of NLRP3 inflammasomes.

In the present study, we revealed that LSS-mediated IKKε phosphorylation promoted the expression of NLRP3 via activating the downstream transcription factor STAT1, leading to endothelial cell pyroptosis and atherosclerosis (Fig. 6). This novel finding declares the pro-pyroptotic effect of LSS-triggered IKKε activation and provides new clues to investigate the mechanisms of atherogenesis and the development of atherosclerosis.

Atherosclerosis is a chronic and complex progressive disease, and endothelial cell dysfunction induced by hemodynamic disturbances is the initial stage in the development of atherosclerosis [13]. As a novel inflammatory cell programmed death, pyroptosis is involved in the development of atherosclerosis [14]. The critical role of NLRP3 inflammasome in endothelial cell pyroptosis has also been proved. NLRP3 drives the activation of the precursor caspase-1, which cleaves the GSDMD and matures the pro-inflammatory factors IL-1β and IL-18, leading to perforation of the cell membrane and release of mature inflammatory factors [15]. Previous studies have revealed that atherogenic risk factors including oxidized low-density lipoprotein (ox-LDL) [16, 17], homocysteine, and nicotine promote endothelial pyroptosis by upregulating or activating NLRP3 via ROS or NFκB pathway [17–19]. A recent study reported that low-shear stress induced ROS accumulation in endothelial cells through upregulation of TET2/SDHB, resulting in the onset of pyroptosis [7]. Consistently, our current study showed that low-shear stress triggered the pyroptosis signaling pathway in HUVECs. However, the mechanism by which LSS triggers endothelial pyroptosis remains unclear due to the lack of research. Our previous studies had confirmed the promoting effects of IKKε in LSS-induced endothelial inflammation and oxidative stress, so we further explored its role and mechanism in LSS-induced endothelial pyroptosis [9, 10].

The non-canonical IκB kinase IKKε is involved in regulating inflammatory signaling pathways by activating downstream molecules NFκB as well as STAT1 in response to pathogenic microbes or oxidative stress [20, 21]. In response to viral infection, IKKε acts as a key molecule in the regulation of innate immunity by activating IRF3 or IRF7. Furthermore, IKKε has been reported to play an important role in chronic diseases such as obesity and type 2 diabetes [22]. In a high-fat diet-induced obesity disease model, IKKε-deficient mice were less susceptible to high-fat-induced chronic liver inflammation and insulin resistance, perhaps due to the involvement of IKKε in fatty acid and glucose metabolism [23, 24]. In a cellular-level study, IKKε knockout endothelial cells were found to exhibit lower levels of IL-18 and VEGF in response to ox-LDL stimulation [25]. Our previous studies found that IKKε mediated LSS-induced endothelial inflammation by activating STAT1 and Akt/IRF3 pathways [9, 10]. Nevertheless, the role of IKKε in LSS-triggered endothelial pyroptosis is still unknown. In the present study, both *in vitro* and *in vivo* experiments indicated that endothelial IKKε silencing reduced LSS-mediated NLRP3 expression, thereby alleviating pyroptosis and aortal arch atherosclerotic lesions. The results above suggest the pro-pyroptotic and pro-atherosclerotic effects of IKKε in the case of low-shear stress.

STAT1 is the earliest identified member of the STATs family, which regulates a variety of biological events such as cell cycle, differentiation, innate or intrinsic immunity, and programmed cell death [26]. Emerging works of research have identified a key role of STAT1 in the development and progression of cardiovascular disease, particularly atherosclerosis [27–29]. STAT1 activation aggravated atherosclerotic lesions and plaque instability via promoting macrophage M1 polarization and pro-inflammatory factors secretion [27, 30]. STAT1 activation in endothelial cells upregulated the expression of inflammatory factor IL-1β and chemokine CX3CL1, triggering atherosclerosis progression [31]. Moreover, STAT1 is reported to be associated with the expression of NLRP3. STAT1 in macrophages exacerbated inflammatory progression by upregulating NLRP3 expression in a mouse model of colitis [32]. Blocking the activation of STAT1 reduced neuronal NLRP3 expression and inflammatory cell infiltration in a mouse traumatic brain injury model [33]. Since our previous study found that IKKε-mediated STAT1 activation was involved in LSS-induced endothelial inflammation and ROS accumulation [9], we further investigated its role in endothelial pyroptosis. Our results showed that STAT1 activation promoted NLRP3 expression and endothelial pyroptosis via binding to the promoter region of NLRP3. In addition, LSS-induced activation of NFκB was not affected by IKKε silencing, suggesting that IKKε does not regulate endothelial cell pyroptosis through the NFκB pathway in response to LSS.

In brief, our data reveal that LSS-induced IKKε phosphorylation activates the downstream transcription factor STAT1 and upregulates NLRP3 expression, ultimately triggering endothelial pyroptosis and atherosclerosis progression. Our innovative study provides new insights into the mechanisms of LSS-triggered endothelial dysfunction, opening up new potential targets for the treatment of atherosclerosis.

### SUPPLEMENTARY INFORMATION

Below is the link to the electronic supplementary material.

Supplementary file1 (DOCX 615 KB)

Supplementary file2 (DOCX 187 KB)

## Data Availability

All the relevant data supporting the findings of this study are available in the article and its Supplementary files, or from the corresponding author on reasonable request.
