# Supplementary material for: Low-Shear Stress Promotes Atherosclerosis via Inducing Endothelial Cell Pyroptosis Mediated by IKKε/STAT1/NLRP3 Pathway
Source: Inflammation. 2024 Feb 5;47(3):1053–66. doi: 10.1007/s10753-023-01960-w (PMC11147929; doi:10.1007/s10753-023-01960-w)
Supplement: Supplementary file 1 — Supplementary file1 (DOCX 615 KB) [file 10753_2023_1960_MOESM1_ESM.docx]

**
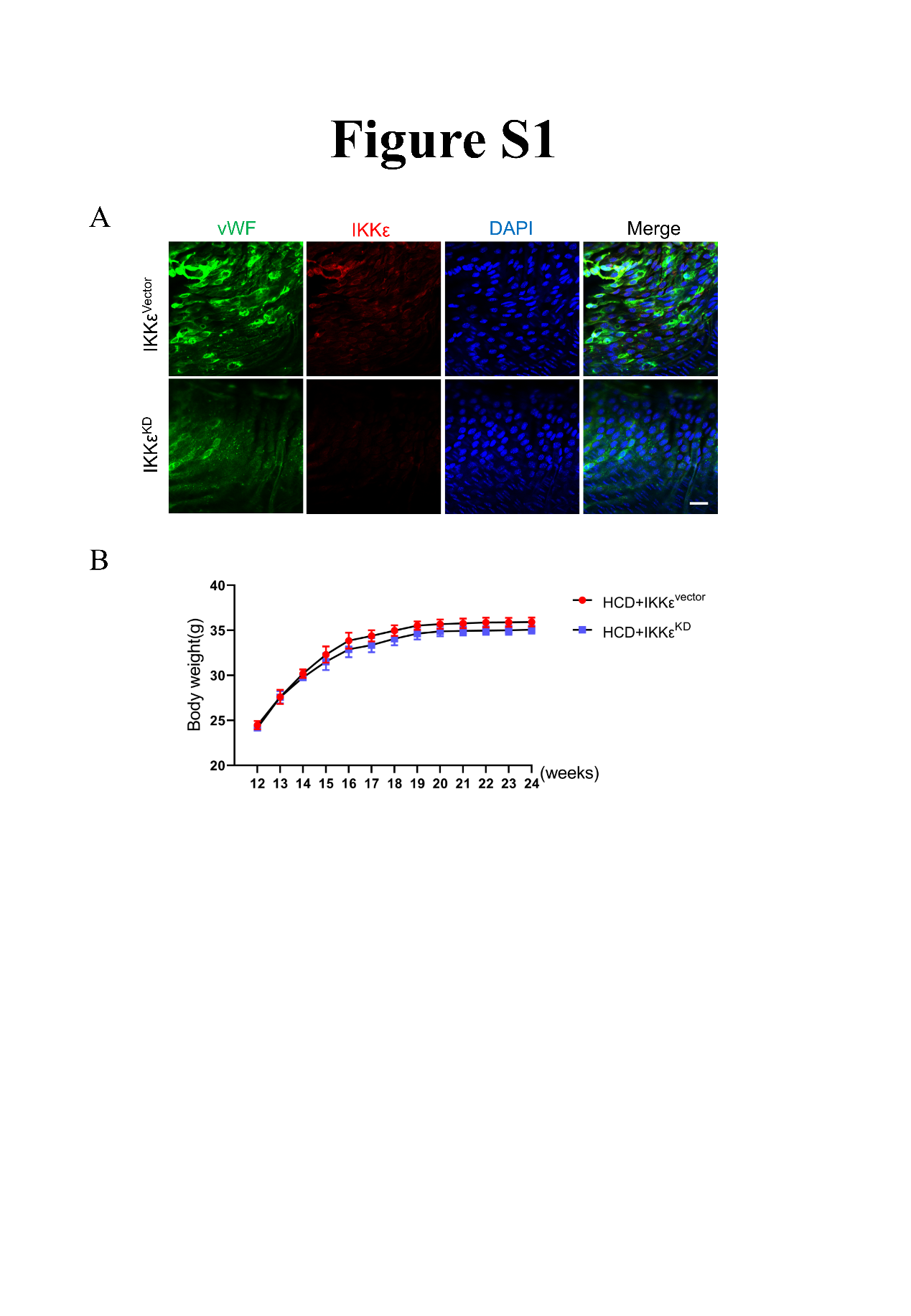
**

**Fig.S1** Establishment of an atherosclerosis model in ApoE^-/-^ mice. (A) Efficiency of endothelial-specific adeno-associated virus knockdown of IKKε detected by Enface staining. vWF: endothelial cells marker. Scale bar=20μm. (B) After the atherosclerosis model was established, the body weight of each mouse in each group was recorded weekly until the model was successfully constructed (*n*=6). The data are presented as mean ± SEM. ^**^*p* < .01
