# Supplementary material for: Low-Shear Stress Promotes Atherosclerosis via Inducing Endothelial Cell Pyroptosis Mediated by IKKε/STAT1/NLRP3 Pathway
Source: Inflammation. 2024 Feb 5;47(3):1053–66. doi: 10.1007/s10753-023-01960-w (PMC11147929; doi:10.1007/s10753-023-01960-w)
Supplement: Supplementary file 2 — Supplementary file2 (DOCX 187 KB) [file 10753_2023_1960_MOESM2_ESM.docx]

| siRNA | | Sequence (5′→3′orientation) | |
| --- | --- | --- | --- |
| Homo-IKKε | Sense | | GUGAAGGUCUUCAACACUACC |
|  | Anti-sense | | UAGUGUUGAAGACCUUCACAG |
| Homo-STAT1 | Sense | | CCUACGAACAUGACCCUAUTT |
|  | Anti-sense | | AUAGGGUCAUGUUCGUAGGTT |
| Negative control | Sense | | UUCUCCGAACGUGUCACGUTT |
|  | Anti-sense | | ACGUGACACGUUCGGA GAATT |

**Supplement Table 1**

**Sequence information of siRNAs used in RNA knock down assay.**

**Supplement Table 2**

**Primers for RT-qPCR assay**

| Primers |  | Sequence (5′→3′orientation) |
| --- | --- | --- |
|  | Forward | CATTCGAACGTCTGCCCTATC |
| 18S |  |  |
|  | Reverse | CCTGCTGCCTTCCTTGGA |
|  |  |  |
|  | Forward | CGTGAGTCCCATTAAGATGGAGT |
| Homo-NLRP3 |  |  |
|  | Reverse | CCCGACAGTGGATATAGAACAGA |
|  |  |  |
|  | Forward | ATTACCCGCCCGAGAAAGG |
| Mus-NLRP3 |  |  |
|  | Reverse | CATGAGTGTGGCTAGATCCAAG |
|  |  |  |

**Supplement Table 3**

**Primers for ChIP assay**

| Primers |  | Sequence (5′→3′orientation) |
| --- | --- | --- |
|  | Forward | TGTCAGAAAACACTGAGTGA |
| Site1 |  |  |
|  | Reverse | GGTCTGTCTGTGCCTCATAC |
|  |  |  |
|  | Forward | TTGGTTCCGATGACTACCAC |
| Site2 |  |  |
|  | Reverse | GGATGGATGAACAAGTGGTT |
|  |  |  |
|  | Forward | TTGGAATTGCCCCCTCCTTG |
| Site3 |  |  |
|  | Reverse | GGTGGGTGAGAGGCACTCGC |
